# Comparison of the Results of a Parkinson's Holter Monitor With Patient Diaries, in Real Conditions of Use: A Sub-analysis of the MoMoPa-EC Clinical Trial

**DOI:** 10.3389/fneur.2022.835249

**Published:** 2022-05-16

**Authors:** Carlos Pérez-López, Jorge Hernández-Vara, Nuria Caballol, Àngels Bayes, Mariateresa Buongiorno, Núria Lopez-Ariztegui, Alexandre Gironell, José López-Sánchez, Juan Carlos Martínez-Castrillo, Alvarez Sauco M, Lydia López-Manzanares, Sonia Escalante-Arroyo, David A. Pérez-Martínez, Alejandro Rodríguez-Molinero, The MoMoPa-EC Research Group

**Affiliations:** ^1^Department of Investigation, Consorci Sanitari de l'Alt Penedès i Garraf, Sant Pere de Ribes, Spain; ^2^Neurology Department, Hospital Universitari Vall D Hebron Neurodegenerative Diseases Research Group, Vall D Hebron Institut de Recerca Universidad Autónoma de Barcelona, Biomedical Research Network Center on Neurodegenerative Diseases (CIBERNED), Instituto de Salud Carlos III, Madrid, Spain; ^3^Department of Neurology, Hospital de Sant Joan Despí Moisès Broggi, Sant Joan Despi, Spain; ^4^Parkinson's and Movement Disorders Unit, Hospital Quirón-Teknon, Barcelona, Spain; ^5^Department of Neurology, Hospital Universitari Mútua Terrassa, Barcelona, Spain; ^6^Department of Neurology, Hospital Universitario de Toledo, Toledo, Spain; ^7^Department of Neurology, Hospital de la Santa Creu i Sant Pau, Barcelona, Spain; ^8^Department of Neurology, Hospital Virgen de la Arrixaca de Murcia, Murcia, Spain; ^9^Movement Disorders and Neurodegenerative Diseases Unit, IRYCIS, Hospital Ramón y Cajal, Madrid, Spain; ^10^Department of Neurology, Hospital General de Elche, Elche, Spain; ^11^Department of Neurology, Hospital Universitario de la Princesa, Madrid, Spain; ^12^Department of Neurology, Hospital de Tortosa Verge de la Cinta, Tortosa, Spain; ^13^Neurology Service, Hospital Universitario 12 de Octubre, Madrid, Spain

**Keywords:** Parkinson's disease, automatic ambulatory monitoring, therapeutic adjustment, wearable sensors, motor fluctuations

## Abstract

**Background:**

For specialists in charge of Parkinson's disease (PD), one of the most time-consuming tasks of the consultations is the assessment of symptoms and motor fluctuations. This task is complex and is usually based on the information provided by the patients themselves, which in most cases is complex and biased. In recent times, different tools have appeared on the market that allow automatic ambulatory monitoring. The MoMoPa-EC clinical trial (NCT04176302) investigates the effect of one of these tools—Sense4Care's STAT-ON—can have on routine clinical practice. In this sub-analysis the agreement between the Hauser diaries and the STAT-ON sensor is analyzed.

**Methods:**

Eighty four patients from MoMoPa-EC cohort were included in this sub-analysis. The intraclass correlation coefficient was calculated between the patient diary entries and the sensor data.

**Results:**

The intraclass correlation coefficient of both methods was 0.57 (95% CI: 0.3–0.73) for the OFF time (%), 0.48 (95% CI: 0.17–0.68) for the time in ON (%), and 0.65 (95% CI%: 0.44–0.78) for the time with dyskinesias (%). Furthermore, the Spearman correlations with the UPDRS scale have been analyzed for different parameters of the two methods. The maximum correlation found was −0.63 (*p* < 0.001) between Mean Fluidity (one of the variables offered by the STAT-dON) and factor 1 of the UPDRS.

**Conclusion:**

This sub-analysis shows a moderate concordance between the two tools, it is clearly appreciated that the correlation between the different UPDRS indices is better with the STAT-ON than with the Hauser diary.

**Trial Registration:**

https://clinicaltrials.gov/show/NCT04176302 (NCT04176302).

## Introduction

Parkinson's disease (PD) is the second most prevalent neurodegenerative disease, after Alzheimer's disease (1), and is characterized by the onset of different motor symptoms, such as tremor, rigidity, and bradykinesia (2). PD has an overall prevalence of approximately 0.5%, which increases to 1% among people aged 65 to 69 years and 1–3% in people aged over 80 (3, 4). Although the neurodegeneration that causes PD affects different regions of the brain, it is pathologically characterized by the loss of nigrostriatal dopaminergic innervation (5).

The treatment of PD is symptomatic, that is, the goal is to alleviate the symptoms that appear throughout the progression of the disease through drugs that, broadly, aim to restore dopamine levels in the striatum (5). Patients generally respond well to treatment during the early stages of the disease, but as PD progresses, the medication lasts for a shorter and shorter time, and motor complications develop that necessitate frequent therapeutic adjustments to control the symptoms adequately (1). Approximately 90% of patients with PD present with motor fluctuations after 10 years (6). Motor fluctuations consists of alternating periods throughout the day when patients present with the symptoms of PD, as if the medication is not effective, and periods when these symptoms disappear to different degrees. The periods when motor symptoms appear are called OFF periods, whereas the periods when they disappear are called ON periods (7). During disease progression, patients tend to present with dyskinesias as an undesired side effect of the medication. Dyskinesias are involuntary movements of the head, trunk, or extremities that usually interfere with patient activity and severely lower the quality of life (8).

The entire spectrum of motor complications is hard to control since these complications typically have a highly variable appearance, fluctuating throughout the day and from day to day. A temporal map of the appearance of different symptoms is very useful for therapeutic adjustment; however, neurologists currently do not have this information and therefore encounter serious difficulties in obtaining good results with medication adjustments. As a general rule, the information neurologists work with on the appearance of symptoms comes from self-reports, either in the form of a diary, in which patients and/or caregivers record their motor state periodically (for ex: Hauser diary), or in retrospective form, in which patients and/or caregivers recount during a visit how they perceive their symptoms throughout the day. Although these methods are the current reference standard, they have biases and errors, since patients often forget to record the motor state, do not adequately recognize the motor state, or confuse the motor symptoms (9).

The recent development of wearable sensors that can monitor fluctuations in motor symptoms opens the door to improving the quantity and quality of this information and thus helps improve the effectiveness of treatments for PD. The objective of this sub-analysis is to study the agreement between the motor fluctuations recorded by patients in a Hauser diary and those detected by a wearable PD Holter monitor. As a secondary objective, this work analyses the correlation between the determinations made by each of these monitoring methods (Hauser diary and the wearable PD Holter monitor) and the score of the Unified Parkinson's Disease Rating Scale (UPDRS), which will be used as an external validation criterion.

MoMoPa-EC is a clinical trial that starts in 2019 and aims to compare the clinical outcomes of PD patients whose motor fluctuations are measured by different methods: a wearable device, patient diary, and information collected during the consultation. In the literature, we can find many published works with Holter monitors for PD, but all of them have been carried out in controlled or laboratory environments and many of them with short evaluation times (10–20). The difference with these studies is that MoMoPa-EC is developed in an environment of routine clinical practice, without controlling the use of the sensor or diary by the researchers but rather letting the patients use them autonomously and without supervision at home after receiving instructions in the doctor's office.

## Methods and Analysis

### Study Design

The present study is a subanalysis of the MoMoPa-EC clinical trial, whose protocol has been published (21). This study was approved by the Research Ethics Committee of Bellvitge Hospital under reference AC012/19. MoMoPa-EC is a randomized, controlled, single-blind clinical trial that aims to compare the clinical outcomes of patients with PD whose motor fluctuations are measured by different methods: wearable (Parkinson's Holter monitor), patient diary (Hauser diary), and information collected during consultation (21). The MoMoPa-EC clinical trial plans to recruit 164 patients with moderate to severe PD and at least 2 h a day in the OFF state. In this trial, after enrolment, all patients fill out a diary of motor fluctuations for 7 days in their homes while wearing a Parkinson's Holter. Then, at the baseline visit, which can take place between 2 and 12 weeks after recruitment, the neurologists participating in the trial administer the UPDRS to all patients.

As detailed in the published protocol, the main objective of MoMoPa-Ec is to evaluate the efficacy of using a Parkinson's Holter compared with traditional clinical practice in terms of Off time reduction with respect to the baseline (recorded through a diary of motor fluctuations). As secondary outcomes, changes in variables related to other motor complications (dyskinesia and FoG), quality of life, autonomy in activities of daily living, adherence to the monitoring system and number of doctor-patient contacts will be analyzed (21). The work presented now is a sub-analysis that make a comparison between the Hauser diary and the sensor output. This analysis was not previously carried out within the objectives of MoMoPa-EC.

In the present subanalysis, the data collected during the monitoring of the patients since enrolment (Parkinson's Holter and Hauser diary) and the scores of the UPDRS administered at the baseline visit were used. Symptom monitoring is repeated during the follow-up of the patients in MoMoPa-EC, but the follow-up data were left out of this subanalysis since they may be influenced by a learning effect through which, with each repetition of symptom monitoring, patients learn to fill out the Hauser diary better, which would have a secondary effect on the agreement between the two monitoring methods.

The neurologists participating in the study explained to all patients how to fill out the Hauser diary. To do this, the neurologists followed a common procedure that consisted of showing instructional videos to patients, which showed examples of motor fluctuations. All baseline Hauser diaries were reviewed by a team of researchers, and the entries that presented problems (lack of information, confusing information, or inconsistencies) were discarded. It was then discussed with the researcher in charge whether the patient would be asked to fill out a diary again (after retraining) or would be excluded. Additionally, patients whose baseline Hauser diaries did not report 2 or more hours on the OFF state per day were excluded (21).

In the MoMoPa-EC trial, the commercial device STAT-ON, manufactured by Sense4Care SL (www.sense4care.com), was the Parkinson's Holter used. This medical device is designed to monitor motor fluctuations and activity in Parkinson's patients on an outpatient basis. The Holter records motor fluctuations during activities of daily living (11), in addition to dyskinesia, bradykinesia, and freezing-of-gait episodes (11, 12, 16, 20). Holter data are stored in the device's internal memory, and users (patients or neurologists) can download them to any mobile phone that has the application provided by the manufacturer installed. This application produces portable document format (PDF) reports that show the data obtained from the patient during the monitored time, including the number of freezing-of-gait episodes detected and the percentages of time in the ON state, the OFF state, and an INTERMEDIATE state. The STAT-ON device is based on the gait bradykinesia for the determination of motor states, the INTERMEDIATE state is when, based on self-adjusted thresholds, the bradykinesia is being detected but not enough to determine an OFF state. For a more in-depth definition of the motor states go to the reference (10). The Parkinson's Holter should be used for at least 3 days for calibration reasons and does not have an upper limit of use (it can be used indefinitely). The manufacturer recommends using the Holter device for 7 days to capture specific changes in motor manifestations and the daily routine, which often occur on weekends.

### Participants

The target population of the MoMoPa-EC study is PD patients with motor fluctuations that are difficult to control. The neurologists participating in the study, who belong to 40 different health centers in Spain, select the participating patients from among those who are being followed up at their outpatient clinics. According to the intended clinical use of the Parkinson's Holter, neurologists are advised to offer the device to patients who could benefit from daily monitoring of their motor symptoms to better control them. It is planned for the trial to include 164 patients who meet all the following inclusion criteria: (1) idiopathic PD according to the clinical criteria of the Brain Bank of the United Kingdom (22), (2) moderate to severe disease (Hoehn & Yahr stage ≥2 in the OFF state) (23), and (3) presence of motor fluctuations, with at least 2 h/day in the OFF state. To be included in the study, patients who have been informed about its objectives have to agree to participate voluntarily and sign a written consent form. Patients who cannot walk independently or who have Hoehn & Yahr stage 5, patients participating in another clinical trial, patients with acute intercurrent disease, patients with psychiatric or cognitive disorders that prevent them from participating in the trial (Mini-Mental State Examination score <24) (24), and patients who have difficulty understanding the study processes (including the ability to correctly fill out the Hauser diary) are excluded. Patient recruitment for the trial began in November 2019 and is ongoing.

**Figure 1:**
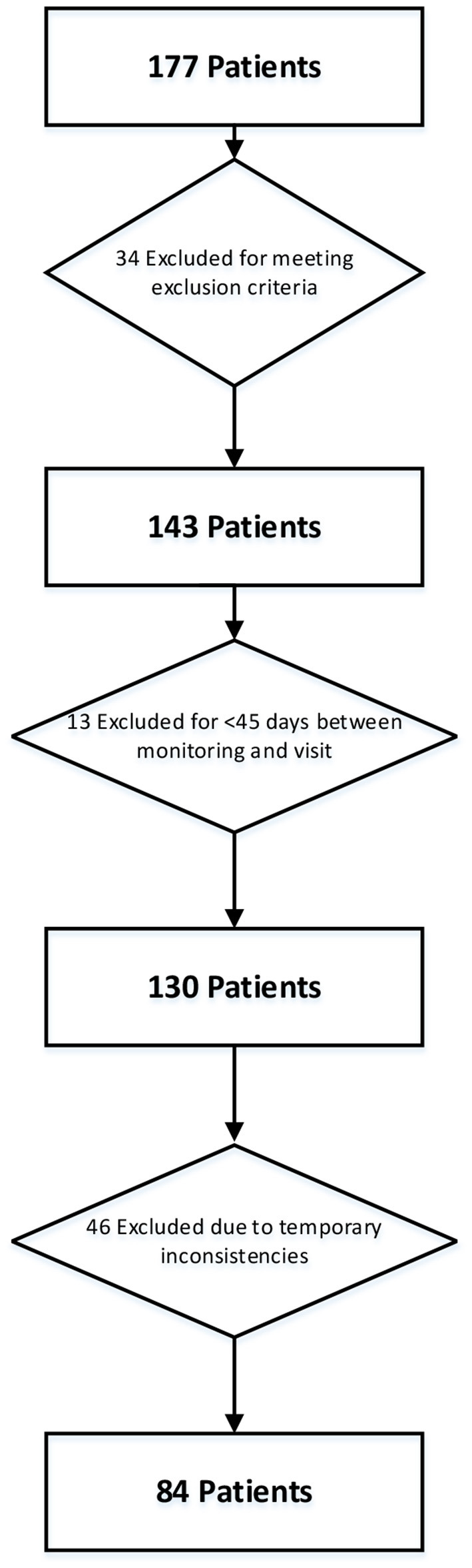
Diagram of the patient selection process for the subanalysis.

Data from 177 patients were available for this sub-analysis, of which 34 were excluded because they did not meet the MoMoPa-EC inclusion criteria. In this subanalysis, only those patients in whom the difference between the baseline visit (where the UPDRS was administered) and the monitoring with STAT-ON were not greater than 45 days were included. This condition was not contemplated in the MoMoPa-EC design but it is necessary for this sub-analysis and, for this reason, 13 patients had to be excluded. Finally, in this sub-analysis it only makes sense to compare those records in which the Hauser diaries and sensor were applied at the same time. This was not a condition in the MoMoPa-EC study either, and it was the main cause of exclusion for this sub-analysis, since monitoring with the Hauser diary and the sensor were not coincident in time in the case of 46 patients. This study was finally carried out in a total of 84 patients who fulfilled all the conditions. Figure 1 shows a diagram illustrating the patient selection process.

### Data Analysis

Only the periods of time when the patients were simultaneously monitored by the two instruments (Parkinson's Holter and Hauser diary) were compared. The Parkinson's Holter, similarly to the Hauser diary, collects data with a temporal frequency of half an hour. In the Hauser diary, the patient can mark four motor states (OFF, ON without dyskinesias, ON with non-problematic dyskinesias, and ON with problematic dyskinesias) and sleeping. The Holter, in addition to identifying the times when the patient experiences dyskinesias, offers three motor states: OFF, ON, and INTERMEDIATE. The INTERMEDIATE state is a state in which the patient has a decrease in stride fluidity, but not as severely as in the OFF state. The Holter also collects different data on other PD symptoms, such as stride fluidity (associated with gait bradykinesia) and the number of freezing-of-gait events, in addition to physical activity parameters, such as the number and length of steps and the accelerometer signal magnitude area (SMA). The Holter bases the detection of motor states on the analysis of gait and, more specifically, on the analysis of stride fluidity; this measurement, which is a continuous variable, is the basis for the detection of OFF states (17–19).

The disparity in detectable states between the two instruments makes it necessary to modify their measurements to be able to compare them. In the STAT ON, the variables of the ON and OFF motor states are presented on the one hand and the dyskinesia as independent variable on the other hand. In order to make an adequate comparison, two analogous variables have been generated with the data from Hauser's diaries, separating the consideration of motor status and the appearance of dyskinesia in Hauser's diary. On the one hand, in Hauser's diary all the ON states have been aggregated together, regardless of whether they presented dyskinesias or not. On the other hand, we have consider as dyskinesia any hour of the Hauser diary marked as dyskinesia in order to compare them with the STAT-ON variable of dyskinesia. In the Holter data, any state that is not ON was considered an OFF state; in other words, the intermediate state was added to the OFF state. In this way, three comparable variables were obtained between the two instruments: OFF, ON, and dyskinesia.

Only data corresponding to periods of time when the two instruments were capturing data were used. For example, on a day in which there were readings from the two instruments, only the time since the second instrument was started (in the case of the Hauser diary, the time of the first reading) until the first instrument stops was considered. Any day that did not have at least 8 h monitored by both instruments was discarded from the analysis. For the analysis, the hours in ON, OFF, and dyskinesias of each day were added and divided by the time monitored that day, yielding the percentage of time in each of the states per monitoring time. Last, the mean of this percentage over all valid monitoring days was calculated. The rest of the continuous variables are expressed as the mean of all the values collected on the valid days.

In the statistical analysis, the parameters obtained by both methods were compared using the intraclass correlation coefficient (ICC) to determine the degree of agreement between the different instruments. For this calculation, the mean mixed-effects model was selected, given that the patient population was a random sample of the total population, whereas the evaluators (in this case the Hauser diary and the Holter) were the totality of the evaluators.

To find correlates of the different parameters of the UPDRS, all possible parameters of the Hauser diary and the Holter were analyzed separately through Spearman correlation. The different UPDRS scores used were the sum of the dyskinesia section of part IV of the UPDRS, the sum of part III of the UPDRS, the sum of part II of the UPDRS, and factor I (bradykinesia/gait). Factor I, described by Stebbins et al. (25) include the sum of: (i) Speech, (ii) Facial expression, (iii) Arise from chair, (iv) Posture, (v) Gait, (vi) Postural stability and (vii) Body bradykinesia.

The ICC calculations were performed in Python (v3.7.1) using the programming library Pingouin 0.3.12. The Spearman correlations were calculated in Python (v3.7.1) using the programming library SciPy 1.5.2.

## Results

Of the 84 patients selected for this analysis, 48 were men and 36 were women. Table 1 shows the mean and median of age, Hoehn & Yahr stage, UPDRS-II and UPDRS-III overall scores, the dyskinesia score of UPDRS-IV and finally OFF, ON and dyskinesia hours from Hauser Diary of the participating patients.

**Table 1:** Patient data.

**Variable**	**Mean**	**Std dev**	**Median**	**IQR**
Age	65.5	9.8	65	15.3
H&Y	2.5	0.4	2.5	1
UPDRS-II	12.5	5.8	12	8
UPDRS-III	21.3	9.7	21.5	16
UPDRS-IV (Dyskinesias)	1.5	1.7	1	2
Factor I	6.0	3.8	5.5	3
OFF hours (7 days Hauser)	30.2	16.4	29	18.8
ON hours (7 days Hauser)	67.1	18.2	67.25	23.6
Hours with dyskinesias (7 days Hauser)	10.6	16.0	1.5	19

*(i) H&Y: Hoehn & Yahr stage, (ii) UPDRS-II: Unified Parkinson's disease Scale part II, (iii) UPDRS-III: Unified Parkinson's disease Scale part III, (iv) UPDRS-IV: the sum of dyskinesia score of Unified Parkinson's disease Scale part IV, (v) Factor I: sum of Speech, Facial expression, Arise from chair, Posture, Gait, Postural stability and Body bradykinesia questions from Unified Parkinson's disease Scale part III, (vi) OFF Hours: sum of OFF hours in 7 days of Hauser diary monitoring, (vii) ON Hours: sum of ON hours in 7 days of Hauser diary monitoring, (viii) Hours with Dyskinesias: sum of Dyskinesia hours in 7 days of Hauser diary monitoring*.

The ICC between the recordings of the Parkinson's Holter and Hauser diary was 0.57 (0.3–0.73) for the percentage of daily time in the OFF state, 0.48 (0.17–0.68) for the percentage of daily time in the ON state, and 0.65 (0.44–0.78) for daily time with dyskinesias. Table 2 shows the Spearman correlations of the different parameters.

**Table 2:** Spearman correlations.

	**Dairy parameter that**	**Rho**	***p*** **value**	**Holter parameter that**	**Rho**	***p*** **value**
	**best correlates**			**best correlates**		
UPDRS-II	Hours ON noDysk	−0.21	0.077	Mean fluidity	−0.42	<0.001
UPDRS-III	Hours On	−0.16	0.19	Mean fluidity	−0.4	<0.001
UPDRS-IV	Hours Dysk	0.57	<0.001	Mean hours dysk	0.47	<0.001
Factor I	Hours On	−0.24	0.16	Mean fluidity	−0.63	<0.001

*(i) UPDRS-II: Unified Parkinson's disease Scale part II, (ii) UPDRS-III: Unified Parkinson's disease Scale part III, (iii) UPDRS-IV: the sum of dyskinesia score of Unified Parkinson's disease Scale part IV, (iv) Factor I: sum of Speech, Facial expression, Arise from chair, Posture, Gait, Postural stability and Body bradykinesia questions from Unified Parkinson's disease Scale part III, (v) Hours ON noDysk: sum the hours without dyskinesia from 7 days Hauser Diary, (vi) Hours ON: sum the hours in ON from 7 days Hauser Diary, (vii) Hours Dysk: sum the hours with dyskinesia from 7 days Hauser Diary, (viii) Mean Fluidity: Mean of fluidity in the days monitored with STAT-ON. (ix) Mean Hours Dysk: Mean of the hours of dyskinesia detected in the days monitored with STAT-ON*.

## Discussion

A moderate ICC was found between the Hauser diary and the Holter data. The correlation of these methods with the UPDRS was low, although it was higher in the case of the Holter than the diary.

In previous studies using the STAT-ON, much higher correspondence was reported between the motor state identified by the Holter and that reported in patient diaries. Previous studies have found, for example, that the sensitivity and specificity of the Holter in detecting the motor state recorded in patient diaries were higher than 90% (10, 11), and the correlation between the Holter data and the UPDRS-III was greater than 0.7 (*n* = 75), even reaching 0.8 in the case of factor I of the UPDRS-III (*n* = 12). The notable differences between our results and previous ones are mainly due to the form and time of implementation of the validation instruments. In previous studies in which the patient diary was used, it was filled out after the patients had received exhaustive specific training by the researchers, while MoMoPa-EC aims to reflect normal clinical practice, so the training is not as rigorous. In addition, patients who completed diaries in the previous studies had a telephone follow-up every 2 h to encourage the use of the diary and clear up any doubts, in addition to a daily face-to-face visit to review the diary entries; none of this is done in the MoMoPa-EC trial. Additionally, in the present study, 45 calendar days could pass between the monitoring of symptoms by Holter or diary and the administration of the UPDRS (although there were no changes in medication between the two times). In contrast, previous studies administered the UPDRS at the same time as the monitoring, which lasted a maximum of 4 h, so this is another substantial methodological difference. On the other hand, in another study carried out with the STAT-ON under real conditions, correlations with the UPDRS were presented practically the same as those obtained in this sub-analysis. Specifically, in this work, a correlation of −0.67 (*p* < 0.001) of Fluidity with factor I of the UPDRS is presented, while in our correlations a −0.63 (*p* < 0.001) was reached (13).

A study with a device much like the STAT-ON, such as the Parkinson's KinetiGraph (PKG; Global Kinetics Corporation), reported a moderate correlation (0.64) in 34 patients with longer monitoring, but in a controlled environment (14). In a study with Kinesia motion sensor units (Great Lakes NeuroTechnologies), Pulliam et al. (15) reported a correlation of 0.81 with the UPDRS-III score, but in a laboratory environment, with a short evaluation time and coincident with the administration of the UPDRS. Again, the difference from these studies is found in the environment in which the MoMoPa-EC clinical trial is developed, an environment of routine clinical practice, without controlling the use of the sensor or diary by the researchers but rather letting the patients use them autonomously and without supervision at home after receiving instructions in the doctor's office. Under these conditions, we interpret the ICC values obtained between both monitoring methods, which in another context would be considered moderate, as a very positive result.

The lack of supervision of MoMoPa-EC study can be interpreted as a limitation when assessing the correlation of STAT-ON with patient diaries. However, in previous publications we have extensively analyzed the correlation of the sensor with the diaries, in a much more controlled situation. This controlled situation is not the one that occurs in clinical practice, and in this study we have had the opportunity to analyze the correlation in real conditions, which is a novel approach and, as expected, the correlation is lower.

It is also worth mentioning that the detection of motor states in the Holter is associated with walking and/or the appearance of dyskinesias. This scheme, which results in a more robust detection, is unable to deliver a diagnostic on the motor status during those periods of time when the patient is not walking and does not have dyskinesias. The diary instead requires frequent manual entry by the patient, and therefore there are many times when the patient does not make records. Thus, the two methods lack data at different times and for different reasons, which limits the degree of agreement between them. On the other hand, the non-motor off states, which are reflected in Hauser's diary, do not always have a correspondence with the motor symptoms, which are what the Holter detects. This fact is another clear limitation for the comparison between these two instruments (26, 27).

In general, the correlation sensor data with the UPDRS scores were better than those of the diary. This was probably because the UPDRS evaluates symptoms in terms of frequency of their appearance and severity, as does the Holter, but in the Hauser diary, only the frequency, not the intensity, of symptoms is recorded.

In contrast, the UPDRS-IV score had a greater correlation with the hours of dyskinesia recorded in the diary than with the hours of dyskinesia recorded by the Holter. This might be explained by the fact that both in the UPDRS-IV and in the diary, it is the patient who reports the symptom, and this symptom is an involuntary movement that often goes unnoticed by the patient. This opens the possibility that there are dyskinesias detected by the Holter that are not reported by the patient when the UPDRS-IV is administered or that are not recorded in the Hauser diary.

In the case of UPDRS-II, the correlation with the diary was very low, the time in the ON state without dyskinesia being the parameter that best correlated. The correlation with the mean fluidity, a Holter parameter, was much higher. This parameter is clearly indicative of the ability of the patient to move and therefore their ability to perform tasks of daily living.

The correlations for UPDRS-III indicate that the parameter that was best correlated with the diary is the hours in the ON state; the fewer the hours in ON, the worse the UPDRS-III score. This correlation is much lower than that between the UPDRS and the gait fluidity parameter of the Holter (16), which is a parameter that analyses the variations in the fluidity of the stride, throughout the monitoring period. The better correlation of this parameter with the UPDRS is consistent with the study by the creators of the Holter, which reported that this parameter has a high correlation with factor I of the UPDRS-III (language, facial expression, arising from a chair, posture, gait, postural stability, bradykinesia, and hypokinesia) and more specifically with gait (16).

Given that the intraclass correlation and the correlations found were moderate or low, a larger sample size would have been necessary to obtain narrower confidence intervals of the estimates. The study of the correlations was also limited by the time elapsed between the monitoring measurements and the administration of the UPDRS. Although medication changes were not allowed during this time according to the clinical trial protocol, it is enough time for the motor manifestations to change spontaneously, making it more difficult to see a good correlation between the measurements performed. Additionally, we do not consider it a limitation but rather a strength that the data for the primary comparison were collected autonomously by the patients, without any supervision. The use of the Holter and the diary was similar to that of daily clinical practice, so the moderate ICCs found reflect the agreement of these instruments under real conditions of use.

The new wearable tools for monitoring motor symptoms in PD that are becoming available on the market are very promising and have great potential to change the way in which patients with PD are evaluated. One of the great barriers to the implementation of these tools is professionals' lack of confidence in them. They lack confidence because there is no standard reference of quality with which to compare them: the Hauser diary has many biases and errors and a completely different time frequency than the new tools. We hope studies such as MoMoPa-EC will help us move past the Hauser diary and will impart confidence in professionals to use these new tools.

## Data Availability Statement

The original contributions presented in the study are included in the article/supplementary material, further inquiries can be directed to the corresponding author/s.

## Ethics Statement

Ethical approval for this study has been obtained from the Hospital Universitari de Bellvitge Ethics Committee. The patients/participants provided their written informed consent to participate in this study.

## MoMoPa-EC Research Group

This research is being conducted by the “Monitoring Parkinson's patients Mobility for therapeutic purposes” (MoMoPa) Research Group, which includes, in addition to the authors of this papers: Anna Planas-Ballvé: Hospital de Sant Joan Despí Moisès Broggi; Pau Pastor, Ignacio Alvarez: Hospital Universitari Mútua Terrassa; Mª Isabel Morales Casado: Hospital Universitario de Toledo; Gema Sánchez: Hospital Universitario Ramón y Cajal; María Asunción Ávila Rivera: Hospital General de l'Hospitalet; Anna Prats: Terapia Integral Uparkinson; María Álvarez Saúco: Hospital General Universitario de Elche; Álvaro Sánchez-Ferro, Antonio Méndez Guerrero: Hospital Universitario 12 de Octubre; Elisabet Franquet Gomez: Hospital Sant Camil; Laura Muñoz-Delgado, Daniel Macías-García, Silvia Jesús, Astrid Adarmes-Gómez, Pablo Mir: Instituto de Biomedicina de Sevilla, Hospital Universitario Virgen del Rocío/CSIC/Universidad de Sevilla; José Mª Salom Juan, Antonio Salvador Aliaga: Hospital Clínico de Valencia; Silvia Martí Martínez, Carlos Leiva Santana: Hospital General de Alicante; Victor M. Puente Pérez, Irene Navalpotro Gómez: Hospital del Mar; Sara Lucas del Pozo: Hospital Vall d'Hebron; Lydia Vela Desojo: Hospital Universitario Fundación Alcorcón; Antonio Koukoulis Fernández: Mª Gema Alonso Losada, Hospital Álvaro Cunqueiro; Mª Esther Cubo Delgado: Hospital Universitario de Burgos; Jon Infante Ceberío, María Sierra Peña, Isabel González Aramburu, Mª Victoria Sánchez Peláez: Hospital Universitario Marqués de Valdecilla; Marina Mata Álvarez-Santullano, Carmen Borrúe Fernández, Mª Concepción Jimeno Montero: Hospital Universitario Infanta Sofía; Mª José Gómez Heredia, Francisco Pérez Errazquin, Lina Carazo Barrios, Clínico Virgen de la Victoria; Alfredo López López: Hospital Royo Villanova; Mª Pilar Solís Pérez, Hospital de Llíria; Rubén Alonso Redondo, Jessica González Ardura: Hospital Univ Lucus Augusti; Javier Ruiz Martínez, Ana Vinagre Aragón, Ioana Croitoru: Hospital Universitario Donostia; Pilar Sánchez Alonso, Elisa Gamo Gonzalez, Sabela Novo Ponte: Hospital Universitario Puerta de Hierro Majadahonda; Esteban Peña Llamas, Hospital Moraleja; Esther Blanco Vicente, Rafael García Ruiz, Ana Rita Santos Pinto, Marta Recio-Bermejo: Hospital Alcázar de San Juan; Judith Jiménez Veiga: Hospital Virgen de la Arrixaca de Murcia; Teresa Muñoz Ruiz, Lucía Flores García: Hospital Regional de Málaga; Rocío García-Ramos, Eva López Valdés: Hospital Clínico San Carlos; Lourdes Ispierto González, Ramiro Álvarez Ramo, Dolores Vilas Rolan: Hospital German Trias i Pujol; Esther Catena Ruiz: Hospital Comarcal de l'Alt Penedès; Ernest Balaguer, Antonio Hernández Vidal: Hospital Universitari General de Catalunya; and Berta Solano Vila, Anna Cots Foraster, and Daniel López Domínguez: Hospital Universitari de Girona Doctor Josep Trueta.

## Author Contributions

CP-L contributed to the statistical analysis plan, study logistics preparation, including software for managing Parkinson's Holter data during the trial, and drafted this article. AR-M conceived and designed the study. JH-V, ÀB, JM-C, and DP-M contributed to the study design. NC, MB, SE-A, AG, AS, NL-A, JL-S, and LL-M have contributed by participating in the study. All authors have read and approved the manuscript.

## Funding

This work was supported by the Instituto de Salud Carlos III (DTS17/00195), the European Fund for Regional Development (‘A way to make Europe'), and AbbVie S.L.U.

## Conflict of Interest

CP-L, AR-M, JH-V, and ÀB were shareholder of Sense4Care the company that markets the tested device. The remaining authors declare that the research was conducted in the absence of any commercial or financial relationships that could be construed as a potential conflict of interest.

## Publisher's Note

All claims expressed in this article are solely those of the authors and do not necessarily represent those of their affiliated organizations, or those of the publisher, the editors and the reviewers. Any product that may be evaluated in this article, or claim that may be made by its manufacturer, is not guaranteed or endorsed by the publisher.
